# Efficacy and Safety of Magnesium Sulfate as an Adjunct to Ropivacaine Wound Infiltration in Thyroid Surgery: A Prospective, Double-Blind, Randomized Controlled Trial

**DOI:** 10.3390/jcm13154499

**Published:** 2024-08-01

**Authors:** Stiliani Laskou, Georgia Tsaousi, Chryssa Pourzitaki, Georgios Papazisis, Isaak Kesisoglou, Konstantinos Sapalidis

**Affiliations:** 1 3rd Surgical Department, School of Medicine, Aristotle University of Thessaloniki, 1st St. Kiriakidi Str, 54636 Thessaloniki, Greece; ikesis@hotmail.com (I.K.); sapalidiskonstantinos@gmail.com (K.S.); 2 Clinic of Anesthesiology and Intensive Care, School of Medicine, Aristotle University of Thessaloniki, 54124 Thessaloniki, Greece; tsaousig@otenet.gr; 3 Department of Clinical Pharmacology, Faculty of Medicine, School of Health Sciences, Aristotle University of Thessaloniki, 54006 Thessaloniki, Greece; chpour@gmail.com (C.P.); papazisg@auth.gr (G.P.)

**Keywords:** ropivacaine, magnesium sulfate, postoperative pain, analgesia, thyroidectomy

## Abstract

**Background/Objective:** Wound infiltration with local anesthetics emerges as a promising modality for postoperative pain alleviation. However, such strategies in neck surgery have not been a well-established practice. To assess wound infiltration with ropivacaine plus magnesium sulfate for pain relief following thyroid surgery. **Methods**: This prospective, double-blind, randomized study enrolled 68 patients who underwent thyroid surgery. Concerning the solution used for surgical wound infiltration, the study participants were randomly allocated into three groups: (1) 100 mg of ropivacaine (Group R); (2) 100 mg of ropivacaine plus magnesium sulfate 10 mg/kg (Group RMg); and (3) normal saline which served as a placebo (Group P). Pain perception both at rest and at movement, was measured using the Visual Analogue Scale (VAS) at 30 min, as well as at 1, 2, 4, 6, 12, and 24 h postoperatively. The total consumption of analgesics in morphine equivalents was also recorded. Moreover, adverse effects and patient satisfaction were recorded. Cortisol, TNF-α, and IL-6 levels were measured 30 min before infiltration and 6 h and 24 h postoperatively. **Results**: Demographics and clinical characteristics were similar between the groups. The VAS scores at rest and during movement were significantly lower in the RMg group compared to the saline or ropivacaine groups. Total analgesic consumption was also significantly lower in the RMg group. No operation-, wound-, or infiltration-related adverse effects were recorded in the study groups. Better overall satisfaction was obtained for the RMg group. **Conclusions**: Ropivacaine plus magnesium sulfate wound infiltration provided better pain control and the analgesic effect was more significant, contributing to effective postoperative analgesia in patients undergoing thyroid surgery.

## 1. Introduction

Thyroid surgery represents a common operation with approximately 93,000 thyroidectomies being performed in the United States each year, while parathyroidectomy is comparatively rare, as the incidence of primary hyperparathyroidism is approximately 25 cases per 100,000 [1]. 

Postoperative pain related to thyroid or parathyroid surgery can be a major challenge for patient care. Pain control after thyroid surgery improves patients’ quality of life and allows a quick return to normal daily activities. Adequate postoperative pain control is customarily managed by a broad spectrum of analgesics, including nonsteroidal anti-inflammatory drugs (NSAIDs) or opioid analgesics, which constitute the top analgesic choices [2,3]. Yet, both regimens are frequently linked to adverse effects.

Local wound infiltration has long been used in general surgery to reduce postoperative pain intensity and minimize the need for analgesics. Evidence indicates that thyroidectomy wound infiltration constitutes an easy-to-implement technique performed by the surgeon himself, while it elongates the time frame until rescue analgesia [4]. In the current clinical practice, magnesium sulfate is widely applied in the perioperative setting for its multifactorial properties, among which it has been shown to mitigate analgesic requirements [5,6,7,8]. Relevant reports indicate that the implementation of wound infiltration with magnesium sulfate and ropivacaine mixture seems to reduce postoperative tramadol requirements after radical prostatectomy [5,6,7,8]. 

To the authors’ knowledge, no studies exist in the current literature investigating the use of magnesium sulfate as an adjunct to local anesthetics for wound infiltration thyroidectomy/parathyroidectomy. Thus, the present study aims to evaluate the effectiveness of wound infiltration with ropivacaine plus magnesium sulfate mixture for postoperative pain control in patients undergoing thyroid or parathyroid surgery, with the safety of this practice constituting the secondary study endpoint.

## 2. Materials and Methods

### 2.1. General Information

After institutional approval was obtained from the Ethics Committee of the School of Medicine of Aristotle University of Thessaloniki (No. 3.468/18-1-2022), all consecutive adult patients of both sexes and the American Society of Anesthesiologists (ASA) classification I–II, scheduled for thyroidectomy/parathyroidectomy from January 2022 to January 2024, were eligible for enrolment in this study. This study was conducted following the Declaration of Helsinki guidelines, while written informed consent was obtained from all patients before study enrollment. The trial was registered in the ClinicalTrials.gov database with the unique reference ID NCT05294393.

The exclusion criteria included the indication for selective lateral neck dissection, previous cervical operation, long-term analgesic drug use, known or suspected allergy to local anesthetics, and a major complication of surgery or anesthesia (major bleeding, allergy to anesthetic products).

### 2.2. Randomization and Masking

Patients were allocated to three groups using a computer randomization list (www.sealedenvelope.com, assessed on 19 January 2022) developed by a person irrelevant to the study protocol. Permuted block randomization was performed using a random number table with equal allocation and a block size of 6. An anesthesia nurse, being unaware of the study protocol, prepared an unlabeled syringe of 15 mL solution, including normal saline which served as a placebo (Group P), ropivacaine 100 mg (Group R), or a mixture of 100 mg ropivacaine plus 10 mg/kg magnesium sulfate (Group RMg). The patients, operating team, and investigator who served as the outcome assessor were blinded to the infiltrated solution. 

### 2.3. Anesthesia and Surgical Procedure

A standardized protocol was applied to all patients. The same anesthesia and surgical team were involved in all procedures. Preoperatively, all patients were premedicated with 0.5 mg alprazolam. In the operating room, venous access with an 18–20 G IV cannula and standard hemodynamic monitoring were applied to all patients. After preoxygenation with 100% oxygen for 3 min, anesthesia induction was established by intravenous 2–2.5 mg·kg^−1^ propofol, 0.2 mg·kg^−1^ cis-atracurium, and target-controlled infusion (TCI) of remifentanil (3 mcg·mL^−1^). Endotracheal intubation was facilitated by spiral endotracheal tubes with inner diameters of 7.5 and 8 mm for females and males, respectively. After anesthesia induction, 8 mg dexamethasone and 40 mg omeprazole were also administered. Maintenance of anesthesia was ensured with desflurane, while remifentanil in TCI mode was applied for intraoperative analgesia. Desflurane was used for its rapid onset and offset, enabling the swift and smooth awakening of the involved patients. A change of 20% in heart rate or mean blood pressure guided TCI rate alterations. 

Patients were placed in the supine position on the operating table, with the neck hyperextended. Through a 4–7 cm skin incision (depending on the size of the thyroid), subplatysmal flaps were created, and strap muscles were separated in the midline and reflected laterally. The middle, superior, and inferior thyroid vessels were then divided. The same steps were repeated for the removal of the contralateral lobe. After complete hemostasis and before wound closure, the surgeon, blinded to the applied medications, soaked a pad with the solution over the anterior face of the trachea, then infiltrated, using a 21-gauge needle, the strap muscles and subcutaneous tissues in both flaps as well as the surgical edges. Extra attention was paid to avoid laryngeal nerve infiltration and thus preserve laryngeal function after surgery. Before surgical wound closure, all patients received 1 g of paracetamol and 4 mg of ondansetron.

Finally, the wound was closed using interrupted 3-0 and 4-0 polyglactin sutures to approximate the strap muscles and the platysma layer, respectively. The skin was closed with 4-0 interrupted polypropylene sutured. Suction drains were not used. All patients were extubated after ensuring enough muscle strength and the ability to follow all commands. Surgical time (incision to wound closure) and awakening time (anesthesia discontinuation to tracheal extubation) were also registered for each participant.

### 2.4. Assessment of Postoperative Pain

All patients were familiarized with a 10 cm Visual Analogue Scale (VAS) preoperatively for pain intensity evaluation. All study participants were followed for 24 h for pain evaluation and adverse effect occurrence. The VAS score at rest and during movement was recorded at 30 min and at 1, 2, 4, 6, 12, and 24 h after surgery completion. In cases of moderate pain at rest (VAS score > 4), 1 gr paracetamol was administered as a rescue analgesic drug, while in cases of severe pain (VAS score > 6), 40 mg parecoxib or 50–100 mg tramadol was administered targeting a VAS < 4. Total analgesic requirements up to 24 h postoperatively were calculated in oral morphine equivalents. Blood samples for the determination of cortisol, TNF-α, and IL-6 levels were collected 30 min before surgical wound infiltration and at 6 and 24 h after surgery completion. Blood samples were placed in dry tubes and centrifuged within 5 min of collection. Thereafter, plasma serum was separated and stored at −70 °C until analyzed. Patient satisfaction was also evaluated with a 5-point scale upon hospital discharge.

### 2.5. Outcomes

The primary study outcome was total analgesic consumption up to 24 h after the study completion. The time to the first analgesic request, pain intensity assessed by the VAS at the predefined time points, magnitude of the inflammatory response, occurrence of adverse effects, and patient satisfaction constituted secondary study endpoints. 

### 2.6. Statistical Analysis

Based on the findings of previous relevant clinical studies in patients undergoing lumbar laminectomy, a sample size of 19 patients was used for each group, with a two-sided alpha of 0.05 and a power of 80% to detect a 24 h reduction in the consumption of rescue analgesics (in oral morphine equivalents) by 30% (standard deviation of 15) between tested groups. Allowing for a 20% drop-out rate, the final study population was set at 68 patients. The sample size analysis was conducted using MedCalc version 16.1.

Data normality was assessed by the Kolmogorov–Smirnoff test. Friedman’s non-parametric test was applied for continuous variable comparisons over different time points (VAS scores at rest and movement), while pairwise comparisons were achieved by the Durbin–Conover test (24 h analgesic consumption). Comparisons by group for each time point were performed by the Kruskal–Wallis test, while the Dwass–Steel–Critchlow–Fligner test was used for pair comparison. Cross-tabulations were applied for qualitative variable comparisons. Data of inflammatory markers were normalized by inverse normal transformation through their fractional rank to facilitate the use of a parametric test. A mixed ANOVA analysis was performed. The sphericity testing criterion with the Mauchly test was not met (*p* < 0.05) and a Greenhouse–Geisser correction was applied. The equality of variances test for equality of covariance in the three patient groups indicated equality of variances at all time points.

The SPSS package (version 29.0) was used for statistical analysis, while a *p*-value < 0.05 was considered statistically significant.

## 3. Results

### 3.1. Clinical and Demographic Characteristics of Study Patients

**Figure 1:**
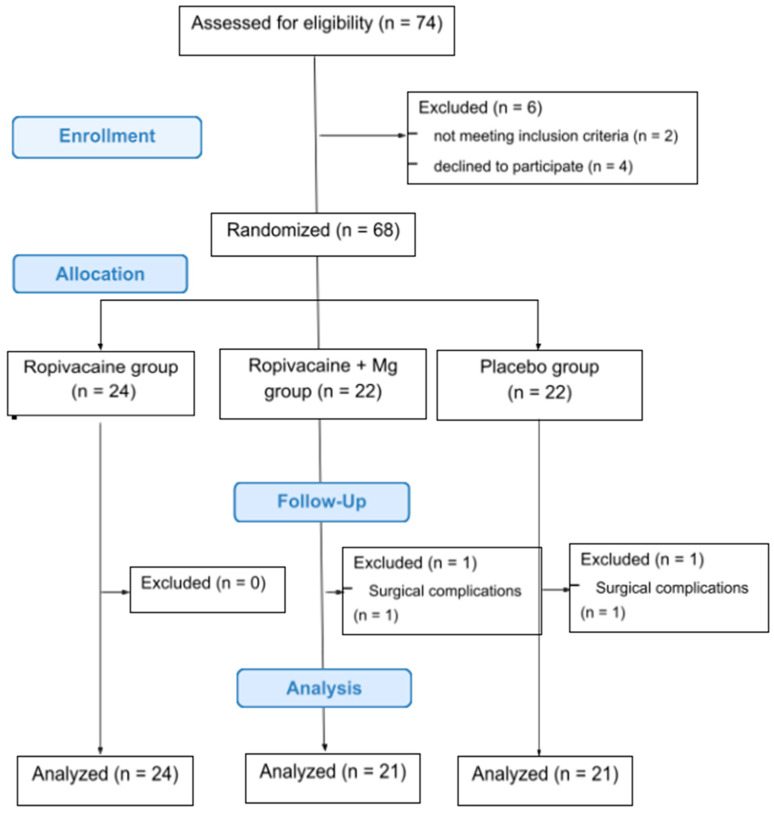
CONSORT diagram. Notes: R, ropivacaine; RMg, ropivacaine plus magnesium sulfate; P, placebo.

From the total of 68 patients initially stratified in this study, two were excluded due to reoperation for severe bleeding control, leaving 66 patients for the final analysis (Figure 1). Demographics and intraoperative data were comparable between study groups (Table 1). There was no statistically significant difference between the groups. As far as the type of surgery was concerned, 41 thyroidectomies were performed for multinodular disease, seven for toxic goiter, six for carcinoma, five for concurrent hyperparathyroidism and goiter, and seven for hyperparathyroidism. 

### 3.2. Total Analgesic Requirements

The total dose of analgesics administered over the 24 h postoperative period varied significantly between the three groups. Patients in Group RMg received less total analgesic requirements than in Group R (7.53 ± 14.55 mg vs. 18.30 ± 13.16 mg, *p* < 0.01, respectively). Patients in the placebo group showed higher analgesic demand than in the RMg and R groups (39.35 ± 19.93 mg vs. 7.53 ± 14.55 mg vs. 18.30 ± 13.16 mg, *p* < 0.001). 

Patients in Group P received their first analgesic drug within the first postoperative hour, while the time to first analgesic request was significantly elongated in the R and RMg groups (Table 2). The time for analgesic demand was significantly lower in the RMg group compared to other groups and in Group R compared to the placebo (χ^2^ (2,66) = 29.42, *p* < 0.001).

### 3.3. Pain Evaluation

The VAS score at rest of all study patients decreased after thyroidectomy at all time points. Higher pain intensity was observed during the first two postoperative hours. Statistical significance was noted when comparing the 1st to 2nd, 4th to 6th, and 6th to 8th postoperative hours. 

**Figure 2:**
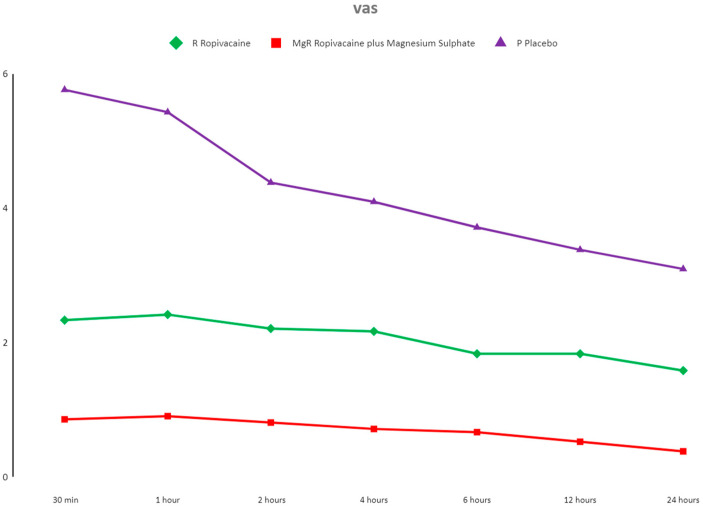
Postoperative Visual Analog Score (VAS) at rest of the three groups presented as the mean and standard deviation. NOTES: All non-parametric Dwass–Steel–Critchlow–Fligner pairwise comparisons were statistically significant at a *p* < 0.001 level. The VAS score at movement of all study patients was increased during the first postoperative hour and decreased afterwards. Statistical significance was noted except for when comparing the 30 min results to the 1st and 2nd h, 2nd to 4th h, and 12th to 24th h.

Comparison between groups revealed significant differences at all timeframes (χ^2^ (6) = 126.34, *p* < 0.001). Analysis showed that the R group obtained lower VAS scores than the placebo at all time intervals. It was also revealed that when magnesium was added to the mixture, the analgesic effect was notably improved compared to ropivacaine alone or the placebo at all time points (Figure 2). 

**Figure 3:**
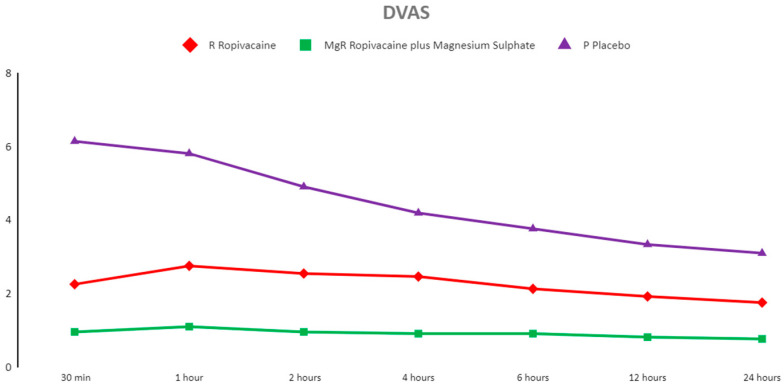
Postoperative Visual Analog Score during movement (DVAS) of the three groups presented as the mean and standard deviation. NOTES: All non-parametric Dwass–Steel–Critchlow–Fligner pairwise comparisons were statistically significant at a *p* < 0.001 level.

Comparison between groups revealed significant differences at all timeframes (χ^2^ (6) = 133.10, *p* < 0.001); ropivacaine showed lower VAS scores compared with the placebo). However, when magnesium was added in the mixture, the analgesic effect was significantly improved. Group RMg showed the lowest VAS scores at all timeframes followed by Group R (Figure 3).

### 3.4. Inflammatory Marker Release

**Figure 4:**
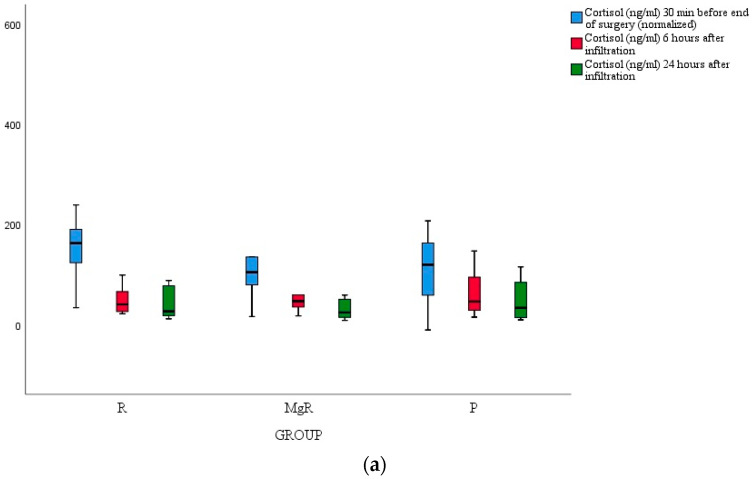

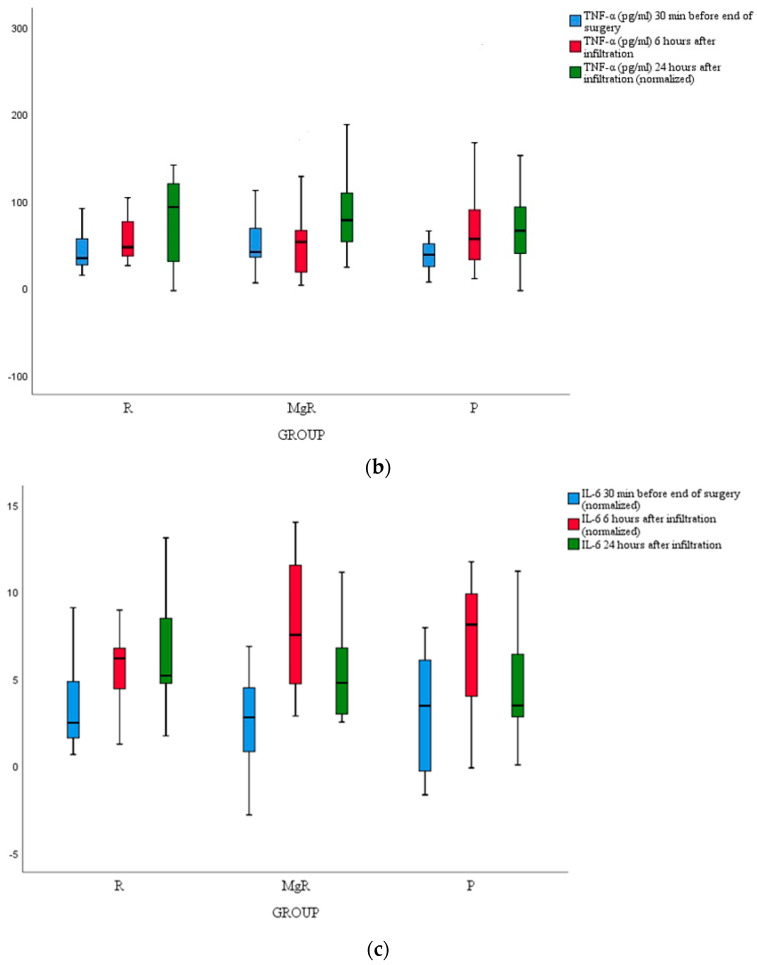
(**a**) Cortisol serum concentration at each time point of assessment; (**b**) TNF-α serum concentration at each time point of assessment; (**c**) IL-6 serum concentration at each time point of assessment.

No statistically significant difference between groups was detected for cortisol, IL-6, and TNF-a levels (Figure 4a–c).

### 3.5. Postoperative Complications

A sore throat was the most commonly reported adverse effect, yet no between-group difference was detected. However, the incidences of nausea and vomiting were similar between the treatment groups (Table 3).

No immediate or short-term cases of mortality were encountered, while wound complications at the injection sites were absent in the study group. Regarding local infiltration agents, no patient developed complications after infiltration. 

### 3.6. Patients’ Satisfaction

Based on the median overall satisfaction score based on a 5-point scale, pairwise comparisons revealed a statistically significant difference (chi-square (2,66) = 16.00, *p* < 0.001). Patients in Group RMg (5(1)) reported better overall satisfaction than patients in the placebo group (3 (1.5)), while a marginal statistical difference was found between patients in the R group (4, (1.75)) and patients in the RMg group.

## 4. Discussion

Postoperative discomfort following thyroid surgery is elicited by multiple causes. Incisional pain especially in the first postoperative day is the major component of this discomfort [9]. The complexity of surgical maneuvers, odynophagia due to endotracheal intubation, and neck soreness caused by the hyperextension position are also incriminated [10]. In the context of Enhanced Recovery After Surgery (ERAS) protocols, which aim to reduce the hospital length of stay while providing faster recovery and functional restoration of the patient, the implementation of a structured analgesic protocol is of paramount importance [11,12]. 

Pain after thyroid surgery is poorly assessed by many physicians, misjudging the small incision. Although these procedures are characterized by a short duration, surgery-related pain is maximized one hour postoperatively and starts to decline 3 h later [11]. Postoperative pain may delay the patient’s discharge as well as return to everyday life.

Pain relief is most commonly achieved by administering NSAIDs and/or opioid analgesics. However, NSAIDs have been associated with potential cardiovascular events, postoperative bleeding, and renal impairment [13]. Meanwhile, opioids are linked to side effects such as sedation, dizziness, nausea, vomiting, constipation, physical dependence, tolerance, and respiratory depression [14].

Based on the fact that the main innervation of the thyroidectomy field originates from the superficial branches of the cervical plexus, regional techniques such as local anesthesia and bilateral superficial and/or deep cervical plexus block could represent a promising method. Local wound infiltration bears the advantage of being a safe and easy-to-implement procedure. It seems that thyroidectomy wound infiltration elongates the timeframe until rescue analgesia. Local anesthetics used for thyroidectomy wound infiltration include the amides bupivacaine, lidocaine, and ropivacaine [9]. 

In our trial, we tried to achieve a long-lasting infiltrative technique by supplementing ropivacaine with magnesium sulfate. Magnesium sulfate reduces NMDA’s receptor binding capacity, preventing central sensitization to a peripheral pain stimulus while preventing the established hyperalgesia [14]. It is even hypothesized to antagonize opioid-induced hyperalgesia as the analgesic effect appears to be better when remifentanil is administered in the anesthetic protocol rather than desflurane [15]. The effects of magnesium sulfate on postoperative pain and opioid consumption have been studied in recent years in a variety of surgical procedures. It has been administered by various routes, including oral, intravenous, intrathecal, and epidural. 

Regarding wound infiltration, magnesium sulfate has been applied, alone or as an adjunct, to prostatectomy, caesarian section, lumbar discectomy, and inguinal hernia repair procedures. In laparoscopic prostatectomy cases, wound infiltration with magnesium achieved a superior analgesic effect compared to the placebo, while in inguinal hernia repair, magnesium was less effective compared to local anesthetic infiltration at reducing opioid requirements [15,16]. Similarly, in pediatric adenectomy, the combination of magnesium with levobupivacaine or ropivacaine reduced postoperative pain and the incidence of laryngospasm compared to the administration of levobupivacaine or ropivacaine alone [17,18]. However, different results were obtained by Sun et al. They assumed that the addition of epinephrine to their solution masked the analgesic effect of magnesium [19].

Notably, in lumbar discectomy procedures, a superior nociceptive effect was attributed to the combined administration of ropivacaine or bupivacaine plus magnesium sulfate [20,21]. More recently, Dave et al. applied the combination of bupivacaine and magnesium sulfate in rectal procedures [22]. Such operations require a deep level of anesthesia since the anorectal region is innervated from both the autonomic nervous system and the animal nervous system, while postoperatively, patients experience severe pain and discomfort. It was found that during the first 24 h, combined administration led to superior results in terms of pain management, less opioid consumption, and pain during defecation. 

The supplementary use of magnesium sulfate in periarticular injection drug mixtures after total arthroplasty procedures has also provided promising findings in terms of early postoperative pain control [23,24]. Noteworthy enough is that no adverse effect was observed in any of the local infiltration studies with magnesium sulfate.

Ropivacaine is a long-acting amide local anesthetic, which reversibly binds and inactivates sodium channels in the open state, inhibiting sodium ion influx in nerve fibers and blocking the propagation of action potentials. Compared to other local anesthetics, it is less likely to penetrate large myelinated motor fibers, acting selectively on the nociceptive A, B, and C fibers. Manufactured as a pure S(−) enantiomer, it is characterized by significantly less cardiotoxicity and neurotoxicity [25,26]. 

Thyroidectomy wound infiltration with ropivacaine has been studied in a few randomized clinical trials. Motamed et al. [27] compared thyroidectomy wound infiltration at the end of surgery with ropivacaine vs. placebo and they observed a significant reduction in pain scales as well as opioid consumption during the patient’s stay in the recovery room but not in the ward. Their observation was attributed to the low ropivacaine dose (20 mg) used for wound infiltration. Comparing local infiltration with ropivacaine (75 mg), bupivacaine (50 mg), and a placebo, Ayman et al. [11] observed a reduction in postoperative pain with ropivacaine but not with bupivacaine. This effect, however, was significant only during the first postoperative hour [11]. Contrary to previous findings, Miu et al. [9] could not prove any significant analgesic benefit with ropivacaine infiltration (75 mg) or a placebo at the end of thyroid surgery. 

It appears that the efficacy of ropivacaine is present but limited when given alone. Both the efficacy and the duration of the method depend on the surgical incision length, as well as the topical agent used [4]. In the same clinical setting, the use of NSAIDs as adjuncts to ropivacaine infiltration promoted an enhanced analgesic effect [12,25,28]. 

In the present study, we attempted to evaluate the analgesic efficacy of the tested infiltrative agents by assessing the magnitude of the inflammatory markers’ release, namely cortisol, TNF-α, and IL-6, but we failed to detect any notable difference between study groups. A plausible explanation for this finding could be the fact that inflammatory response modulation was a secondary study endpoint, and presumably, our study was underpowered for this effect. Over and above, it appears that thyroidectomy-induced stress response is trivial considering that the average VAS score at rest and movement was maintained at clinically acceptable levels throughout the study course [9,29,30].

Several limitations could be acknowledged in the present study. First, a possible drawback could be the absence of serum magnesium level determination, as well as the recording of subsequent magnesium-related adverse effects. Second, intraoperative opioid titration was guided by hemodynamic alterations. Although the implementation of nociception monitoring seems to reflect intraoperative stimuli slightly better than traditionally used parameters and contribute to the quantification of the patient’s physiological pain response, to date, none among the commercially available devices has shown a convincing and clinically relevant benefit for its routine use [31,32,33,34,35]. 

Third, although we applied a randomization process in our study methodology, a higher intraoperative remifentanil consumption was recorded in the placebo group, yet the difference was not statistically significant. This finding could imply the development of a hyperalgesic effect in this group of patients. Fourth, pain assessment was achieved by a subjective scale which could be associated with bias since patients may have interpreted a sore throat or neck pain as incisional pain. Lastly, by conducting a single-center study, our findings reflect the effect of this practice in a defined population subjected to a standardized surgical practice. 

## 5. Conclusions

In conclusion, local wound infiltration with magnesium sulfate as an adjunct to ropivacaine can enhance the postoperative analgesic effect in comparison to either ropivacaine or a placebo following total thyroidectomy, further demonstrating an advanced safety profile. Future large-scale studies are mandatory to enlighten the role of magnesium sulfate as an adjunct analgesic regimen for wound infiltration in this clinical setting.

## Figures and Tables

**Table 1:** Demographic and intraoperative characteristics of the patients in the ropivacaine (R), ropivacaine plus magnesium sulfate (RMg), and placebo (P) groups. NOTES: R, ropivacaine; RMg, ropivacaine plus magnesium sulphate; P, placebo; M, male; F, female; kg/m^2^, kilogram per square meter; cm, centimeter; μg, microgram; min, minute.

	Group R	Group RMg	Group P
Sex (M:F)	4:20	5:16	5:16
Age (years) BMI (kg/m^2^)	54.78 ± 15.08	51.48 ± 15.56	62.14 ± 10.29
BMI (kg/m^2^)	27.49 ± 4.44	28.06 ± 6.25	27.52 ± 5.70
Hamilton Anxiety Scale	13.17 ± 5.44	14.05 ± 4.19	12.86 ± 3.55
Duration of Surgery (min)	108.54 ± 38.16	123.52 ± 27.11	125.05 ± 32.23
Anesthesia Interruption to Extubation Time (min)	5.75 ± 1.87	6.19 ± 3.37	6.43 ± 2.40
Incision Length (cm)	4.39 ± 1.05	4.50 ± 0.62	4.54 ± 0.72
Remifentanil TCI (μg)	795.96 ± 287.16	729.96 ± 389.40	1064.36 ± 457.94

**Table 2:** Time to first analgesic request and analgesic consumption expressed in morphine equivalents.

	Group R	Group RMg	Group P
1st analgesic requirement (min)	140.60 ± 127.56 *	184.13 ± 131.58 *^	53.75 ± 60.62
Total analgesic requirements in morphine equivalents (mg)	18.30 ± 13.16 *	7.53 ± 14.55 *^	39.35 ± 19.93

Notes: * *p* < 0.001 between treatment groups vs. control. ^ *p* < 0.01 between Mg and RMg groups.

**Table 3:** Postoperative complications. NOTES: PONV, postoperative nausea and vomiting.

	Group R	Group RMg	Group P
PONV	2 (3.3%)	2 (3.3%)	3 (4.9%)
Shiver	1 (6.1%)	1 (1.5%)	0 (0%)
Cough	4 (6.1%)	2 (3%)	2 (3%)
Sore Throat	9 (13.6%)	6 (9.1%)	9 (13.6%)

## Data Availability

The original contributions presented in the study are included in the article, further inquiries can be directed to the corresponding author.

## References

[1] Terris D.J., Snyder S., Carneiro-Pla D., Iii W.B.I., Kandil E., Orloff L., Shindo M., Tufano R.P., Tuttle R.M., Urken M. (2013). American Thyroid Association statement on outpatient thyroidectomy. Thyroid.

[2] Bianchini C., Malago M., Crema L., Aimoni C., Matarazzo T., Bortolazzi S., Ciorba A., Pelucchi S., Pastore A. (2016). Post-operative pain management in head and neck cancer patients: Predictive factors and efficacy of therapy. Acta Otorhinolaryngol. Ital..

[3] Nabata K.J., Guo R., Nguyen A., Osborn J.A., Wiseman S.M. (2022). Superiority of non-opioid postoperative pain management after thyroid and parathyroid operations: A systematic review and meta-analysis. Surg. Oncol..

[4] Laskou S., Tsaousi G., Pourzitaki C., Loukipoudi L., Papazisis G., Kesisoglou I., Sapalidis K. (2023). Local Wound Infiltration for Thyroidectomized Patients in the Era of Multimodal Analgesia. Medicina (Kaunas).

[5] Tauzin-Fin P., Sesay M., Svartz L., Krol-Houdek M.C., Maurette P. (2009). Wound infiltration with magnesium sulphate and ropivacaine mixture reduces postoperative tramadol requirements after radical prostatectomy. Acta Anaesthesiol. Scand..

[6] Eldaba A.A., Amr Y.M., Sobhy R.A. (2013). Effect of wound infiltration with bupivacaine or lower dose bupivacaine/magnesium versus placebo for postoperative analgesia after cesarean section. Anesth. Essays Res..

[7] Kundra S., Singh R.M., Singh G., Singh T., Jarewal V., Katyal S. (2016). Efficacy of Magnesium Sulphate as an Adjunct to Ropivacaine in Local Infiltration for Postoperative Pain Following Lower Segment Caesarean Section. J. Clin. Diagn. Res..

[8] Donadi P., Moningi S., Gopinath R. (2014). Comparison of bupivacaine anδ bupivacaine plus magnesium sulphate infiltration for postoperative analgesia in patients undergoing lumbar laminectomy: A prospective randomised double-blinded controlled study. J. Neuroanaesth. Crit. Care.

[9] Miu M., Royer C., Gaillat C., Schaup B., Menegaux F., Langeron O., Riou B., Aubrun F. (2016). Lack of Analgesic Effect Induced by Ropivacaine Wound Infiltration in Thyroid Surgery: A Randomized, Double-Blind, Placebo-Controlled Trial. Anesth. Analg..

[10] Lacoste L., Thomas D., Kraimps J.L., Chabin M., Ingrand P., Barbier J., Fusciardi J. (1997). Postthyroidectomy analgesia: Morphine, buprenorphine, or bupivacaine?. J. Clin. Anesth..

[11] Ayman M., Materazzi G., Bericotti M., Rago R., Nidal Y., Miccoli P. (2012). Bupivacaine 0.5% versus ropivacaine 0.75% wound infiltration to decrease postoperative pain in total thyroidectomy, a prospective controlled study. Minerva Chir..

[12] Li X., Yu L., Yang J., Tan H. (2019). Multimodal analgesia with ropivacaine wound infiltration and intravenous flurbiprofen axetil provides enhanced analgesic effects after radical thyroidectomy: A randomized controlled trial. BMC Anesthesiol..

[13] Ghlichloo I., Gerriets V. (2023). Nonsteroidal Anti-Inflammatory Drugs (NSAIDs) [Updated 2023 May 1]. StatPearls.

[14] Benyamin R., Trescot A.M., Datta S., Buenaventura R., Adlaka R., Sehgal N., Glaser S.E., Vallejo R. (2008). Opioid complications and side effects. Pain Physician.

[15] Lee C., Song Y.-K., Jeong H.-M., Park S.-N. (2011). The effects of magnesium sulfate infiltration on perioperative opioid consumption and opioid-induced hyperalgesia in patients undergoing robot-assisted laparoscopic prostatectomy with remifentanil-based anesthesia. Korean J. Anesthesiol..

[16] Razavi S.S., Peyvandi H., Jam A.R.B., Safari F., Teymourian H., Mohajerani S.A. (2015). Versus Bupivacaine Infiltration in Controlling Postoperative Pain in Inguinal Hernia Repair. Anesth. Pain Med..

[17] Karaaslan K., Yilmaz F., Gulcu N., Sarpkaya A., Colak C., Kocoglu H. (2008). The effects of levobupivacaine versus levobupivacaine plus magnesium infiltration on postoperative analgesia and laryngospasm in pediatric tonsillectomy patients. Int. J. Pediatr. Otorhinolaryngol..

[18] Derbel R., Achour I., Thabet W., Chakroun A., Zouch I., Charfeddine I. (2022). Addition of magnesium sulfate to bupivacaine improves analgesic efficacy after tonsillectomy: A randomized trial and a CONSORT analysis. Eur. Ann. Otorhinolaryngol. Head Neck. Dis..

[19] Sun J., Wu X., Zhao X., Chen F., Wang W. (2015). Pre-emptive peritonsillar infiltration of magnesium sulphate and ropivacaine vs. ropivacaine or magnesium alone for relief of post-adenotonsillectomy pain in children. Int. J. Pediatr. Otorhinolaryngol..

[20] Sane S., Mahdkhah A., Golabi P., Hesami S.A., Kazemi Haki B. (2020). Comparison the effect of bupivacaine plus magnesium sulfate with ropivacaine plus magnesium sulfate infiltration on postoperative pain in patients undergoing lumbar laminectomy with general anesthesia. Br. J. Neurosurg..

[21] Hazarika R., Parua S., Choudhury D., Barooah R.K. (2017). Comparison of Bupivacaine Plus Magnesium Sulfate and Ropivacaine Plus Magnesium Sulfate Infiltration for Postoperative Analgesia in Patients Undergoing Lumbar Laminectomy: A Randomized Double-blinded Study. Anesth. Essays Res..

[22] Dave S., Gopalakrishnan K., Krishnan S., Natarajan N. (2022). Analgesic Efficacy of Addition of Magnesium Sulfate to Bupivacaine in Wound Infiltration Technique in Perianal Surgeries. Anesth. Essays Res..

[23] Wang Q., Zhao C., Hu J., Ma T., Yang J., Kang P. (2023). Efficacy of a Modified Cocktail for Periarticular Local Infiltration Analgesia in Total Knee Arthroplasty: A Prospective, Double-Blinded, Randomized Controlled Trial. J. Bone Jt. Surg..

[24] Zhao C., Wang L., Chen L., Wang Q., Kang P. (2023). Effects of magnesium sulfate on periarticular infiltration analgesia in total knee arthroplasty: A prospective, double-blind, randomized controlled trial. J. Orthop. Surg. Res..

[25] George A.M., Liu M. (2023). Ropivacaine. [Updated 2023 Jul 31]. StatPearls.

[26] Graf B.M., Abraham I., Eberbach N., Kunst G., Stowe D.F., Martin E. (2002). Differences in cardiotoxicity of bupivacaine and ropivacaine are the result of physicochemical and stereoselective properties. Anesthesiology.

[27] Motamed C., Merle J.C., Combes X., Yahkou L., Saidi N.E., Degranges P., Dhonneur G. (2007). Postthyroidectomy pain control using ropivacaine wound infiltration after intraoperative remifentanil: A prospective double blind randomized controlled study. Acute Pain..

[28] Karamanlioglu B., Turan A., Memis D., Kaya G., Ozata S., Ture M. (2005). Infiltration with ropivacaine plus lornoxicam reduces postoperative pain and opioid consumption. Can. J. Anaesth..

[29] Kilbas Z., Mentes M.Ö., Harlak A., Yigit T., Balkan S.M., Cosar A., Öztürk E., Kozak O., Tufan C.T. (2015). Efficacy of wound infiltration with lornoxicam for postoperative analgesia following thyroidectomy: A prospective, randomized, double-blind study. Turk. J. Med. Sci..

[30] Yücel A., Yazıcı A., Müderris T., Gül F. (2016). Comparison of lornoxicam and low-dose tramadol for management of post-thyroidectomy pain. Agriculture.

[31] Ledowski T., Ang B., Schmarbeck T., Rhodes J. (2009). Monitoring of sympathetic tone to assess postoperative pain: Skin conductance vs surgical stress index. Anaesthesia.

[32] Jensen E.W., Valencia J.F., López A., Anglada T., Agustí M., Ramos Y., Serra R., Jospin M., Pineda P., Gambus P. (2014). Monitoring hypnotic effect and nociception with two EEG-derived indices, qCON and qNOX, during general anaesthesia. Acta Anaesthesiol. Scand.

[33] Jakuscheit A., Weth J., Lichtner G., Jurth C., Rehberg B., von Dincklage F. (2017). Intraoperative monitoring of analgesia using nociceptive reflexes correlates with delayed extubation and immediate postoperative pain: A prospective observational study. Eur. J. Anaesthesiol..

[34] Ledowski T. (2019). Objective monitoring of nociception: A review of current commercial solutions. Br. J. Anaesth..

[35] Hirose M., Okutani H., Hashimoto K., Ueki R., Shimode N., Kariya N., Takao Y., Tatara T. (2022). Intraoperative Assessment of Surgical Stress Response Using Nociception Monitor under General Anesthesia and Postoperative Complications: A Narrative Review. J. Clin. Med..

